# The Role of PI3K in Met Driven Cancer: A Recap

**DOI:** 10.3389/fmolb.2018.00086

**Published:** 2018-10-24

**Authors:** Alexia Hervieu, Stéphanie Kermorgant

**Affiliations:** ^1^Signal Transduction and Molecular Pharmacology Team, Cancer Therapeutics Division, Institute of Cancer Research, Sutton, United Kingdom; ^2^Spatial Signalling Team, Centre for Tumor Biology, Barts Cancer Institute, Queen Mary University of London, London, United Kingdom

**Keywords:** Met, PI3K, Akt, mTOR, cancer, signaling

## Abstract

The Receptor Tyrosine Kinase (RTK) Met, overexpressed or mutated in cancer, plays a major role in cancer progression and represents an attractive target for cancer therapy. However RTK inhibitors can lead to drug resistance, explaining the necessity to develop therapies that target downstream signaling. Phosphatidylinositide 3-kinase (PI3K) is one of the most deregulated pathways in cancer and implicated in various types of cancer. PI3K signaling is also a major signaling pathway downstream of RTK, including Met. PI3K major effectors include Akt and “mechanistic Target of Rapamycin” (mTOR), which each play key roles in numerous and various cell functions. Advancements made due to the development of molecular and pharmaceutical tools now allow us to delve into the roles of each independently. In this review, we summarize the current understanding we possess of the activation and role of PI3K/Akt/mTOR, downstream of Met, in cancer.

## The receptor tyrosine kinase met

Met is the RTK for the hepatocyte growth factor (HGF), which is also called the scatter factor (SF) (Cooper et al., 1984; Bottaro et al., 1991). *In vivo*, HGF-dependent Met signaling controls a complex biological program known as *invasive growth program*. This program is required for many biological processes, such as tissue morphogenesis and homeostasis during embryogenesis (Bladt et al., 1995; Schmidt et al., 1995; Uehara et al., 1995), or wound healing and tissue regeneration during adulthood (Michalopoulos and Defrances, 1997; Chmielowiec et al., 2007). Once Met is activated by HGF binding, Met induces cell migration and cell proliferation and protects cells from apoptosis (Rodrigues et al., 1997; Royal et al., 2000; Trusolino and Comoglio, 2002). Also, Met promotes angiogenesis (Bussolino et al., 1992; Grant et al., 1993). The following references can be referred to for detailed reviews on the topic (Gherardi et al., 2012; Comoglio et al., 2018).

### Met structure and activation

The mature form of Met is a heterodimer consisting of an extracellular alpha chain and a beta chain that spans the membrane. The N-terminus extracellular part of Met constitutes the HGF-binding domain. In addition, the beta chain is composed of a Met transmembrane helix and the intracellular portion (Gentile et al., 2008; Gherardi et al., 2012). The intracellular portion contains three regions: (1) a juxtamembrane segment with a serine residue (Ser 985; Gandino et al., 1994) and a tyrosine (Tyr 1003; Peschard et al., 2001) negatively regulating the receptor; (2) the tyrosine kinase domain including Tyr 1234 and Tyr 1235; (3) the C-terminal region, containing Tyr 1349 and Tyr 1356, a multidocking site involved in signal transduction (Gentile et al., 2008; Gherardi et al., 2012; further described later).

HGF binding to Met induces Met dimerization and stabilization, conferring to Met an active conformation (Gherardi et al., 2003; Furlan et al., 2014). The tyrosines of Met in the kinase domain are autophosphorylated, which is followed by the transphosphorylation of its tyrosines in the multidocking site.

Once activated, Met recruits most of its downstream signals to the multidocking site either directly or indirectly, due to the presence of scaffolding molecules or adaptors (Ponzetto et al., 1994; Furge et al., 2000). This multidocking site, formed of only two tyrosines (Tyr 1349 and Tyr 1356), is unique to the Met receptor and has not been observed in other RTKs. The main Met adaptors include growth factor receptor bound protein 2 (Grb2) (Fixman et al., 1996), sarcoma (Src) homology-2-containing (Shc) (Pelicci et al., 1995), and Grb2-associated binding protein 1 (Gab1). Gab1 can bind Met directly or indirectly through Grb2 (Weidner et al., 1996).

### Met signaling

Downstream of Met, many signaling pathways are activated to induce one cell function, while one signaling pathway can induce many cell functions. Interestingly, recent studies have reported that receptors do not signal only from the plasma membrane, but also post-internalization from endosomes, prior to their degradation in the lysosome (Sadowski et al., 2009; Scita and Di Fiore, 2010). This has been described for several RTKs including epidermal growth factor receptor (EGFR) (Wiley and Burke, 2001; Wang et al., 2002; Miaczynska, 2013), and more recently, Met (Kermorgant et al., 2004; Kermorgant and Parker, 2008). We have discovered that two gain of functions Met mutants, M1268T and D1246N, initially identified in human papillary renal carcinomas, are oncogenic not only because they are highly activated, but also because they signal from endosomes (Joffre et al., 2011). In many cancers, Met is mostly overexpressed rather than mutated. We have shown that, in several cancer cell lines expressing endogenous nonmutated Met, HGF stimulation triggers a rapid internalization of Met (Kermorgant et al., 2003, 2004; Kermorgant and Parker, 2008; Ménard et al., 2014; Barrow-McGee et al., 2016). Interestingly, Met remains bound to HGF and activated on endosomes. Furthermore, the optimal activation by Met of signals, such as ERK1/2, STAT3, and Rac1 requires an intact endocytosis machinery (Kermorgant et al., 2004). Met was shown to signal in several types of endosomes: the early endosome, the late endosome, and a novel endosome decorated by LC3 that we named “Autophagy Related Endomembrane, ARE” (Kermorgant et al., 2004; Kermorgant and Parker, 2008; Joffre et al., 2011; Ménard et al., 2014; Barrow-McGee et al., 2016).

Why and how Met signaling operates on endosome is not clearly understood. Our studies so far indicate that signaling on endosomes promotes signal duration. We have shown that Met needs to traffic to perinuclear endosomes/late endosomes to allow sustained activation and nuclear accumulation of the transcription factor STAT3 (Kermorgant et al., 2003, 2004) and sustained activation of the Rho-GTPase Rac1 (Ménard et al., 2014). Interestingly, the mechanisms involved are different. It was noticed that Met is a weak activator of STAT3, as STAT3 becomes rapidly dephosphorylated by cytoplasmic phosphatases. However, the perinuclear Met localization allows a local activation of STAT3 and rapid nuclear accumulation, thus avoiding dephosphorylation (Kermorgant and Parker, 2008; Mcshane and Zerial, 2008; Rosse et al., 2010). Although Rac1 can be activated by Met from the early and the late endosomes, the pathway between Met and Rac1 is distinct. Only in the late endosome, Met requires class I PI3K activity and the GEF Vav2 to activate Rac1, leading to a sustained activation, subsequent actin remodeling, and cell migration (Ménard et al., 2014). This suggests that Met accesses specific effectors in specific endosomes, thus providing a platform for specific signaling cascades.

Altogether these studies suggest that Met promotes oncogenic signaling from endosomes and that the pathways and subsequent cell responses are “endosome-type” specific.

## The Pi3K signaling pathway

**Figure 1:**
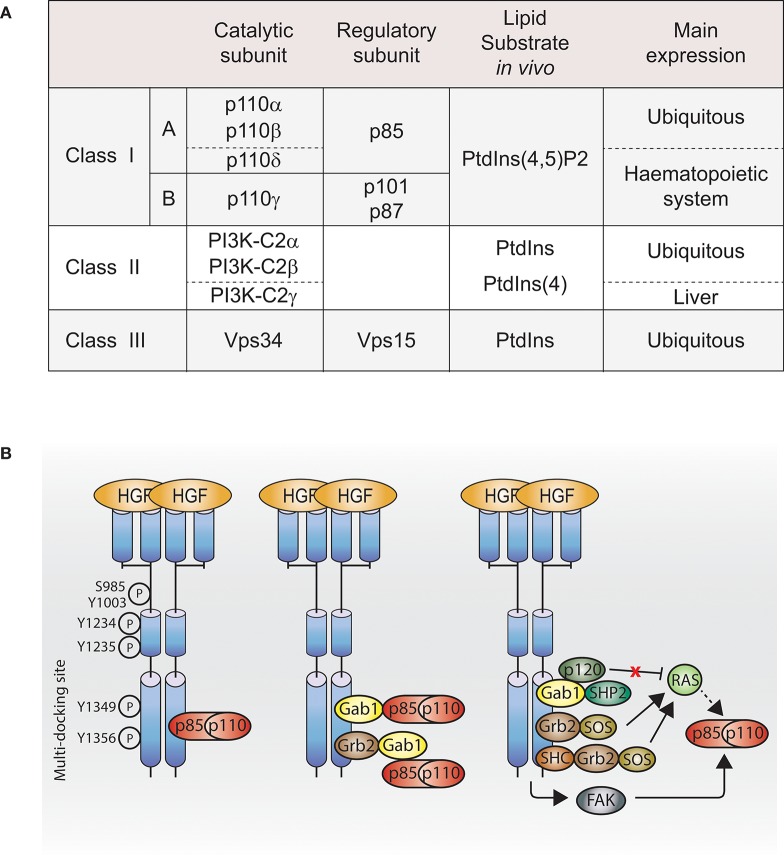
PI3K activation by Met. **(A)** Table of PI3K isoforms and classes. Height PI3K isoforms divided into three classes depending on their lipid substrate, their catalytic subunit and their regulatory subunit. **(B)** Met, directly or indirectly, through adaptors, scaffolding molecules or other downstream signaling molecules, regulates class I PI3K activation. Dashed line: not demonstrated downstream of Met. Ptdlns(4,5)P2, phosphatidylinositol-4,5-bisphosphate; Ptdlns, phosphatidylinositol; Ptdlns4P, phosphatidylinositoi-4-phosphate; HGF, hepatocyte growth factor; PI3K, p110-p85; Grb2, growth factor receptor bound protein 2; She, Sarcoma (SRC) homology-2-containing; Gab1, Grb2-associated binding protein 1; SHP2, Src homology domain-containing 5′ inositol phosphatase 2; p120, p120-ras-GTPase activating protein; SOS, son of sevenless; FAK, focal adhesion kinase.

The phosphatidylinositide 3-kinase (PI3K) enzymes exhibit dual activities that include lipid kinase activity (phosphorylate the 3-hydroxyl group of the inositol ring of their own lipid substrates; Figure 1A) and protein kinase activity (autoregulation; Dhand et al., 1994; Maheshwari et al., 2017), which play a role in many key cell functions, such as cell proliferation, migration, differentiation, survival, and trafficking. The PI3K family contains eight isoforms divided into three distinct classes (I, II, and III; Figure 1A), which may play a role in the specificity of cellular responses.

The largest and the most understood of them is the class I PI3K. Akt and mTOR are the two-most studied indirect effectors of class I PI3K.

### Akt activation

The production of phosphatidylinositol-3,4,5-triphosphate (PIP3) by class I PI3K leads to (1) the recruitment and activation of phosphoinositide-dependent kinase-1 (PDK1) at the plasma membrane; (2) the recruitment of Akt at the plasma membrane; (3) the phosphorylation of the threonine 308 of Akt by activated PDK1; and (4) the phosphorylation of the serine 473 of Akt by mTOR complex 2 (mTORC2) (Sarbassov et al., 2005). These two phosphorylations are necessary for the full activation of Akt. Due to PIP3 localization, it is traditionally thought that Akt signals, when fully activated, from the plasma membrane; however, some recent studies suggest otherwise (see Spatial Signaling of Met and PI3K Pathway).

### MTOR activation

Growth factors stimulate the PI3K/Akt and mitogen-activated protein kinases (MAPKs)/ribosomal s6 kinase (RSK) signaling pathways, which inhibit the tuberous sclerosis 1 (TSC1)/tuberin (TSC2) heterodimer, leading to Ras homolog enriched in brain (Rheb) activation and subsequent activation of mTOR kinase activity in mTOR complex 1 (mTORC1) (Inoki et al., 2002; Potter et al., 2002; Ma et al., 2005). In parallel, and independently of TSC1/2 regulation, Akt can directly inhibit the mTORC1 inhibitor proline-rich Akt substrate of 40 kDa (PRAS40) (Sancak et al., 2007; Vander Haar et al., 2007). Growth factors stimulate mTORC2 activation possibly through PIP3 (Gan et al., 2011), which induces the activation of mTORC2 substrates (Sarbassov et al., 2005). The mechanism by which PIP3 contributes to mTORC2 activation is unknown.

PI3K has multiple effectors independent of Akt (Vanhaesebroeck et al., 2012), and mTOR can be activated by multiple signaling pathways (Laplante and Sabatini, 2012). Moreover, the wide use of compounds referred to as “pan-PI3K inhibitors” may have misled numerous conclusions. Compounds, such as LY294002 and wortmannin inhibit class I and III, but not class II PI3K (Domin et al., 1997; Kong et al., 2010), and inhibit numerous off-targets, including mTOR, depending on the concentration used (Kong et al., 2010). For this reason, in this review, we have only reported studies that have provided evidences not solely based on these inhibitors (LY294002 and wortmannin, often at high concentration), but in conjunction with other tools. The emergence of specific inhibitors targeting PI3K isoforms and PI3K effectors, such as of Akt or mTOR, will improve the knowledge of the individual role of each molecule in the pathway (Rodon and Tabernero, 2017; Janku et al., 2018).

### Interaction of met and class I Pi3K: mechanisms of activation

Studies in the 1990s aimed to identify Met effectors upon Met activation, their direct binding sites, and their molecular adaptors allowing indirect binding. Among the Met effectors discovered, there is PI3K (Graziani et al., 1991). In parallel, in 1990s, PI3K structure and lipid substrates/products were being better understood. Among the three PI3K classes described, the existing literature reports that class I PI3K can act downstream of Met. However, so far, which class I PI3K isoform(s) is/are downstream of Met and whether class II and III PI3K are involved in Met signaling are unknown.

### Direct interaction

Class I PI3K isoforms are able to bind Met directly through the Src homology 2 (SH2) domains present in the class I PI3K regulatory subunits (Ponzetto et al., 1993, 1994), while a direct interaction between Met and class II or class III PI3K is unknown. The class I PI3K regulatory subunit p85 coimmunoprecipitates with phosphorylated Met (Graziani et al., 1991), and can bind synthetic Met peptides phosphorylated on various tyrosines (Y1307, Y1313, Y1349, and Y1356) *in vitro*. In cells, p85 presents a high affinity for Met double-binding site Y1349-Y1356 (Ponzetto et al., 1993; Figure 1B, left panel).

### Indirect interaction

#### Gab1

Class I PI3K can bind Met indirectly through binding the Met adaptor Gab1 (Figure 1B, middle panel; Ponzetto et al., 1994; Maroun et al., 1999). The binding of class I PI3K to Gab1 upon Met activation might be dependent on the integrin alpha3beta1. Upon HGF stimulation, Gab1 and p85 coimmunoprecipitate with Met in immortalized epithelial cells generated from mice expressing wild-type integrin alpha3beta1; however, this was not the case in epithelial cells from mice knocked out for alpha3beta1 (Liu et al., 2009).

#### FAK

Class I PI3K may be activated through focal adhesion kinase (FAK) in mouse inner medullary collecting duct-3 (mIMCD-3) epithelial cells following HGF stimulation (Figure 1B, right panel; Ishibe et al., 2004).

#### Ras

Class I PI3K can be activated directly by the small GTPase Ras pathway (Figure 1B, right panel; Rodriguez-Viciana et al., 1994; Potempa and Ridley, 1998). The catalytic subunits of class I PI3K, p110 subunits, present a Ras-binding domain (RBD), and Ras plays an important role in p110 subunits activation (Suire et al., 2006; Gupta et al., 2007). Met-dependent p110 subunits activation through Ras has not been reported. However, Ras activation by Met is well-described in the context of the MAPK (Potempa and Ridley, 1998). Met activates Ras through several mechanisms: (1) the adaptor Grb2 binds to Met multifunctional docking site and associates with Son Of Sevenless (SOS), a guanine nucleotide exchange factor (GEF) for Ras (Ponzetto et al., 1994); (2) Grb2 associates indirectly with Met via SH2 domain-containing transforming protein (SHC) (Pelicci et al., 1995); (3) Grb2, bound directly to Met, can recruit the adaptor Gab1, which promotes Met-dependent Ras activation by binding the tyrosine phosphatase SHP2, which presumably like for EGFR, dephosphorylates the Gab1 binding site for p120-Ras-GAP leading to the recruitment and inhibition of p120-Ras-GAP, which, when active, inhibits Ras activation (Maroun et al., 2000; Montagner et al., 2005). Thus, future investigations will reveal whether Ras can mediate Met activation of PI3K through such pathways (Figure 1B, right panel).

### Met and Pi3K effectors and their cell functions

PI3K, Akt, and mTOR are involved in various cell functions induced by Met. Cell survival and cell migration are the best described.

### Cell survival

**Figure 2:**
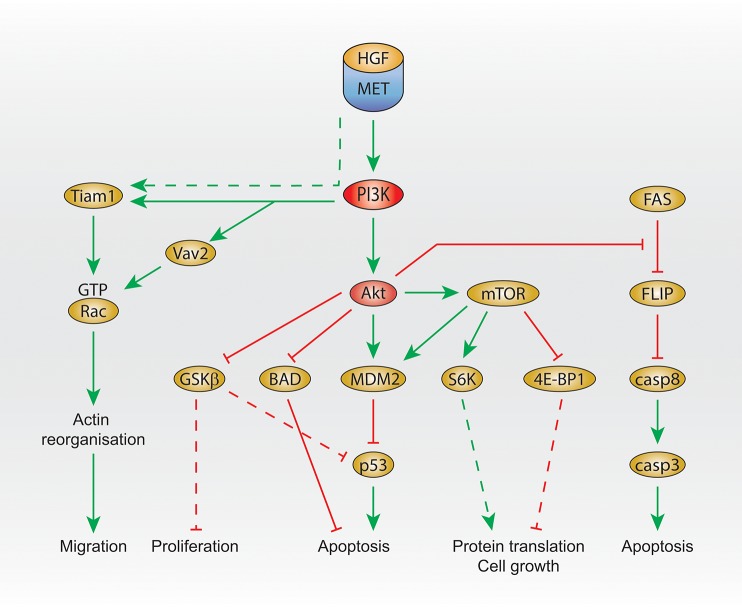
PI3K signaling activated by Met. Summary of Met/PI3K class I downstream signaling, that regulates migration, proliferation, apoptosis, protein translation and cell growth. Dashed line: not demonstrated downstream of Met. HGF, hepatocyte growth factor; PI3K, phosphatidylinositol 3-kinase; GTP, guanosine triphosphate; GSK3 beta, glycogen synthase kinase 3 beta; BAD, Bcl-2 antagonist of cell death; MDM2, mouse double minute 2 homolog; mTOR, mechanistic target of rapamycin; eiF, eukaryotic translation initiation factor; 4E-BP1, 4E–binding protein 1; FLIP, FLICE-like inhibitory protein; casp, caspase 3/8.

In primary embryonic hepatocytes, HGF treatment leads to the activation of Akt and mTOR, and the subsequent inhibition of apoptosis by activating the E3 ubiquitin-protein ligase MDM2, inhibitor of p53 (Figure 2). MDM2 activation is inhibited by the PI3K and mTOR inhibitor LY294002, the Akt inhibitor A-443654, and the mTOR inhibitor rapamycin (Moumen et al., 2007b). Moreover, glycogen synthase kinase 3 beta (GSK3beta) (downstream of Akt) is phosphorylated, which results in its inhibition, leading to p53 inhibition as GSK3beta activation is involved in the activation of p53.

In addition, Akt was shown to inhibit Beclin (Bcl)-2 antagonist of cell death (BAD) upon HGF treatment, leading to the reduction of apoptosis (Figure 2), which is prevented by the PI3K inhibitor wortmannin treatment at low concentration (Liu, 1999).

Another signal that seems to be regulated by Met and PI3K in order to protect cells from apoptosis is FLICE-like inhibitory protein (FLIP). The FLIP inhibits caspase-8, which is involved in apoptosis. Upstream of FLIP, Fas promotes FLIP degradation (Figure 2). In embryonic hepatocyte cells, HGF treatment prevents FLIP degradation, and this is inhibited by treatment with the PI3K and mTOR inhibitor LY294002 or the Akt inhibitor A-443654 (Moumen et al., 2007a).

The signaling pathway NFkappaB, which is involved in numerous cell functions (e.g., survival, proliferation, and morphogenesis), is regulated by Met through PI3K and Akt, leading to apoptosis protection. In DU-145 prostate cancer cells and Madin–Darby canine kidney (MDCK) epithelial cells, HGF induces the activation of the transcription factor NFkappaB. This is inhibited by the expression of a dominant negative form of Akt or overexpression of the phosphatase and tensin homolog deleted on chromosome 10 (PTEN), which converts PIP3 to PIP2, and thus has the opposite function of PI3K. This was mediated by the serine/threonine p21-activated kinase 1 (Pak1), downstream of Akt, and led to the expression of the antiapoptotic factor TNF receptor associated factor 2 (TRAF-2) and calf-intestinal alkaline phosphatase (cIAP) (Fan et al., 2005).

Interestingly, a study suggests distinct roles for PI3K depending on how it is activated by Met (Fan et al., 2001). When Met activates PI3K through Met-Grb2 binding domain, cell survival and Akt phosphorylation are induced. When a mutant of Gab1 that is unable to bind PI3K is overexpressed, there is no effect and Met is still able to induce a similar level of Akt phosphorylation and cell survival. However, when wild-type Gab1 is overexpressed, Met-dependent cell survival and Akt phosphorylation are reduced. The authors concluded that PI3K recruitment through Gab1 downstream of Met is responsible for the redirection of PI3K signaling for other functions than cell survival.

### Cell migration

Studies using the PI3K/mTOR inhibitor LY294002 reported that PI3K is required for Met-dependent Rac activation (Royal et al., 2000; Ishibe et al., 2004; Ménard et al., 2014).

Rac is a member of the small GTPase Rho family (with Cdc42 and Rho) and has 3 isoforms: Rac1 (ubiquitously expressed), Rac2 (mainly expressed in hematopoietic cells) and Rac3 (highly expressed in the brain, and found in many other tissues). Among Rac isoforms, only Rac1 has been described to be downstream of Met and mediates cell scattering through the promotion of actin reorganization (Ridley et al., 1995). Rac binds GTP when activated, and GDP when inactivated. Switching between these two conformations is positively regulated by GEFs and negatively regulated by GTPase Activating Proteins (GAPs) and Guanine nucleotide Dissociation Inhibitors (GDIs). GEFs promote GDP dissociation and GTP association. Their activation can occur directly and indirectly by RTKs (Schiller, 2006). Class I PI3K stimulates the production of phosphatidylinositol-3,4,5-triphosphate (PIP3), leading to binding and activation of a GEF, through its Pleckstrin Homology (PH) domain, resulting ultimately to an increase of Rac activity (Desai et al., 2010).

Interestingly, the GEF T-lymphoma invasion and metastasis (Tiam1) is activated upon HGF stimulation (Singleton et al., 2007) and is required for Met-dependent Rac1 activation (Palamidessi et al., 2008; Joffre et al., 2011; Ménard et al., 2014). Recently, we have shown that, in breast cancer cells, Met-dependent Rac1 activation on the late endosome and subsequent cell migration require the GEF Vav2 and can be inhibited by the PI3K/mTOR inhibitor LY294002. Moreover, downstream of Met, the binding of Vav2 to PIP3 is required for Rac1 activation (Palamidessi et al., 2008; Joffre et al., 2011; Ménard et al., 2014; Figure 2).

### Tissue morphogenesis

HGF has been shown to induce the formation of branched tubules from mammary epithelial cells cultured on Matrigel. This was inhibited by the compounds LY294002 and wortmannin at low concentration (10 nM), indicating the role of PI3K in this mechanism (Niemann et al., 1998). By contrast, alveolar formation was dependent on neuregulin and ErbB2 and components of the ras/MAPK kinase pathway (Yang et al., 1995; Niemann et al., 1998).

### Chromosome instability

PI3K and Akt might be involved in Met-dependent chromosome instability. HeLa cells overexpressing the constitutively active M1268T Met have supernumerary centrosomes and aneuploidy, which is inhibited by the PI3K/mTOR inhibitor LY294002, knockdown of Akt, or overexpression of PTEN (Nam et al., 2010).

### Spatial signaling of met and Pi3K pathway

Increasing amounts of studies suggest the importance of the localization of the receptors and their signals within the cell, such as on various endomembranes, to regulate cell functions (Joffre et al., 2011; Ménard et al., 2014; Barrow-McGee et al., 2016). Recent studies suggest that Met can activate the PI3K pathway from endosomes.

Membrane trafficking (formation, movement, docking, and fusion of vesicles) is a mechanism regulated by the small GTPases Rab. Among them, Rab5 plays an essential role in early endosomes trafficking. The “Adaptor Protein containing PH domain, phosphotyrosine binding (PTB) domain, and leucine zipper motif 1” (Appl1), a Rab5 effector (Miaczynska et al., 2004), is required on endosomes to induce Akt phosphorylation and cell survival in zebrafish (Schenck et al., 2008). In 2010, Tan et al. have generated mice knockout (KO) for Appl1 (Tan et al., 2010). Insulin or EGFR triggered same levels of Akt phosphorylation in wild-type Appl1 and KO Appl1 mouse embryonic fibroblasts (MEF). However, Met-dependent Akt phosphorylation was strikingly reduced in Appl1 KO MEFs as compared to wild-type cells. This reduction was observed in both Akt phospho-sites: serine 473 and threonine 308. The remaining phosphorylation of Akt induced by Met in Appl1 KO MEFs was further reduced by the knockdown of Appl2 (Tan et al., 2010). These results indicate that Met requires Appl proteins to phosphorylate Akt. Moreover, due to Appl1/2 location, and the fact that Appl proteins are effective only when located in endosomes, these results suggest that Met could activate Akt from Appl1 endosomes.

Another study suggesting that PI3K is activated by Met on endosomes was performed in our laboratory. Thus, as mentioned earlier, we have shown that, in MDA-MB-468 cells, Met activates Rac1 on the late endosome via recruitment of the GEF Vav2 and PI3K (Ménard et al., 2014). Confocal live imaging detected the colocalization of fluorescently labeled HGF with Vav2-GFP and with p85alpha-GFP (class I PI3K regulatory subunit) in perinuclear Rab7 positive endosomes. Vav2 mutated in the PH domain (which binds to PIP3, the product of class I PI3K), was unable to mediate Met-dependent Rac1 activation (Figure 2).

Another potential link between Met trafficking and Akt activation was reported in C6 glioma cells, in which the knockdown of Na^+^/H^+^ exchanger (NHE5) increased HGF-induced Met degradation, reducing its recycling back to the plasma membrane as well as Akt phosphorylation (Fan et al., 2016).

Relationships between Met, PI3K/mTOR signaling, and autophagy have recently been reported. Activated Met colocalizes with LC3B (microtubule-associated protein 1A/1B-light chain 3) in various cell lines including breast, lung, and colon cancer cells (Barrow-McGee et al., 2016; Lampada et al., 2017). Although Met-containing autophagy vesicles require further characterization, our results so far suggest that these “autophagy related endomembranes,” or ARE, are distinct from autophagosome and belong to a novel noncanonical autophagy pathway (Barrow-McGee et al., 2016). The ATG5 or beclin1 siRNA knockdown leads to reduced Met-dependent phosphorylation of ERK1/2 (Barrow-McGee et al., 2016) and reduced cell survival in anoikis (Barrow-McGee et al., 2016). In autophagy-proficient colorectal cancer cells, the knockdown of rictor (mTORC2 scaffolding protein) reduced the phosphorylation of Met (Lampada et al., 2017). These results suggest LC3B autophagic vesicles/ARE are novel signaling platforms for Met.

## Targeting met and Pi3K/Akt/mTOR pathway in cancer

The most common way Met becomes oncogenic is through overexpression of Met and/or HGF. Met has been shown to drive many different types of cancer including in lung, liver, stomach, breast, pancreas, brain (Gherardi et al., 2012; Comoglio et al., 2018). Hundreds of Met mutations have also been discovered in all types of cancers, such as breast, gastric, head and neck, liver and lung cancer (Danilkovitch-Miagkova and Zbar, 2002; Gherardi et al., 2012; see COSMIC: the Catalog of Somatic Mutations in Cancer, http://cancer.sanger.ac.uk/cosmic/gene/analysis?ln=MET#dist).

Patients with overexpression of HGF and/or Met, or mutated Met, have a poor prognosis (Jeffers et al., 1998; Gherardi et al., 2012).

Moreover, Met and HGF overexpression have been associated with drug resistance to cancer therapy including against EGFR (Engelman et al., 2007; Bardelli et al., 2013) and Raf (Straussman et al., 2012). Met-mediated resistance to cancer therapy has been observed in several cancer types including breast, lung, pancreatic, and colorectal cancer (Mueller et al., 2010; Bardelli et al., 2013; Hage et al., 2013).

Due to a better understanding of the ligand, the receptor, and activators, many HGF and Met inhibitors have been developed (inhibitors for HGF activators, HGF inhibitors, Met antagonists, and mainly Met kinase inhibitors) and some are being tested in clinical trials for cancer therapy (Luraghi et al., 2012; Maroun and Rowlands, 2014; Zhang et al., 2018). Crizotinib and Cabozantinib, two ATP competitors for multiple tyrosine kinases including Met, have been approved for cancer treatment, while ATP competitors targeting Met only are still in clinical trial, such as Capmatinib, which is in phase II (Wu et al., 2016; Table 1).

**Table 1:** Met, PI3K, and mTORC1 inhibition in cancer therapy.

**Compound**	**Targets**	**Status**
Crizotinib	MET, ALK, ROS1, RON	Approved
Cabozantinib	VEGFR2, MET, RET, KIT, FLTs, TIE2, AXL	Approved
Capmatinib	MET	Phase II
Copanlisib	Pan-class I PI3K	Approved
Idelalisib	p110 delta	Approved
Taselisib	p110 alpha, delta and gamma	Phase III
Sirolimus	mTORC1	Approved
Everolimus	mTORC1	Approved
Temsirolimus	mTORC1	Approved

But RTK (and thus Met) inhibitors can lead to drug resistance in cancer therapy (Engelman et al., 2007; Stommel et al., 2007; Guix et al., 2008; Cepero et al., 2010); it is, therefore, necessary to develop therapies targeting downstream signaling of Met, possibly to be used in combination with Met inhibition.

In cancer or aging, the PI3K signaling pathway is frequently deregulated and PI3K is more active due to mutations or amplification. In solid tumors, these deregulations occur mainly on p110 alpha among all of the PI3K isoforms (Samuels et al., 2004; Vogt et al., 2007). However, overexpression of the other class I PI3K isoforms can also transform cells *in vitro* (Kang et al., 2006). The increase of PI3K activity in cancer is often associated with the altered function of the tumor suppressor PTEN, due to either loss of heterozygosity or mutations. The function of the PIP3 phosphatase PTEN is to antagonize class I PI3K signaling. Mutations/deletion in the PIK3R1 gene, which encodes for 3 species of the p85 regulatory subunit (p85 alpha, p55 alpha, and p50 alpha), have also been found in cancer (Wood et al., 2007; Parsons et al., 2008; Jaiswal et al., 2009). For those reasons, cancer treatments so far have focused on targeting class I PI3K. The pharmacological inhibitors Copanlisib, pan-class I PI3K, and Idelalisib, specific to p110 delta isoform, have been approved for cancer treatment (Furman et al., 2014; Dreyling et al., 2017), while Taselisib, specific to p110 alpha, delta, and gamma isoforms, is in clinical trial phase III (Dickler et al., 2016; Baselga et al., 2017; Table 1). Further reading can be found in the following reviews (Rodon and Tabernero, 2017; Janku et al., 2018).

Deregulations can also occur downstream of PI3K. Indeed, mutations of PDK1, PTEN, or Akt have been discovered in cancer, which affect Akt or mTOR signaling. mTOR is well-known as an indirect PI3K effector involved in mitogenesis. It plays an essential role for numerous cell functions, such as proliferation and cell growth, and its deregulation can lead to tumor growth, angiogenesis, and metastasis (Laplante and Sabatini, 2012). Several rapalogs (mTORC1 inhibitors) have been approved for cancer treatment, such as sirolimus, everolimus, and temsirolimus (Hudes et al., 2007; Motzer et al., 2008; Table 1).

Class I PI3K activation occurring in cancer frequently also results from RTK activation (Moscatello et al., 1998; Moulder et al., 2001; Yakes et al., 2002; Bianco et al., 2003; Engelman et al., 2005; Mellinghoff et al., 2005; Berns et al., 2007; Engelman, 2009). Research is ongoing to test the possible benefit of inhibiting Met or PI3K/Akt/mTOR for cancer therapy. So far, there is no drug/compound available targeting specifically Met and PI3K interaction. Interestingly, Met and PI3K/Akt/mTOR pathways are simultaneously deregulated in various cancers. For example, an increase of Met and Akt phosphorylation has been reported in the PCI-15 radioresistant head and neck cancer cell line (Ettl et al., 2015). The acquired resistance to doxorubicin of the ovarian cancer cell line A2780 appears mediated through Met overexpression. The inhibition of Met and the use of the PI3K/mTOR inhibitor LY294002 repressed the resistance (Jung et al., 2015). In malignant pleural mesothelioma, overexpression of Met, Akt, and mTOR have been demonstrated, and the combination of Met and dual PI3K/mTOR inhibitors showed synergistic effect in reducing mesothelioma cell lines viability and mouse xenografts growth (Kanteti et al., 2014). Similarly, the effect of combined Met and PI3K or mTOR inhibition was evaluated in epitheloid sarcoma cell lines (Imura et al., 2014), and in head and neck cancer cells (Nisa et al., 2017). In both cases, the combination of Met and PI3K or mTOR inhibitors reduced tumor growth *in vivo* better than with a single agent. The level of Met expression and Akt phosphorylation were investigated in human salivary gland tumors and were found to correlate (Vasconcelos et al., 2015).

Thus, assessing PI3K/mTOR expression along with Met expression in cancer samples may provide biomarker value to stratify patients likely to respond to therapies targeting these molecules. Moreover, cotargeting Met and PI3K/mTOR may improve patient outcome.

## Conclusions

The failure of monotherapies, mostly due to drug resistance because of cellular compensations, increases the need for new cancer treatments. Combinatorial targeting of Met and PI3K/Akt/mTOR might help reduce or delay the development of drug resistance. Further research is required to unravel how Met signals to PI3K/mTOR. For example: (i) a better understanding of which PI3K isoform(s) play(s) a role in Met-driven oncogenic cell behavior and (ii) dissecting how Met-PI3K-mTOR signaling orchestrates spatially within cancer cells, will help design such therapies and stratify the patients that will benefit from it. Understanding the role of these key regulators downstream of Met seems essential for the successful development of combinatorial therapies and improve efficacy while reducing toxicity.

## Author contributions

AH suggested the topic and wrote the manuscript. AH and SK edited the manuscript.

### Conflict of interest statement

The authors declare that the research was conducted in the absence of any commercial or financial relationships that could be construed as a potential conflict of interest.
